# Supplementary material for: Deep learning-based idiomatic expression recognition for the Amharic language
Source: PLoS One. 2023 Dec 14;18(12):e0295339. doi: 10.1371/journal.pone.0295339 (PMC10720994; doi:10.1371/journal.pone.0295339)
Supplement: S1 Appendix — “The English translation of the stop words eliminated in this study“. (PDF) [file pone.0295339.s001.pdf]

Appendix

The English translation of the stop words eliminated in this study

All<br>Only<br>One<br>Recent<br>All of them<br>Especially<br>In terms of<br>Bottom<br>Back<br>Main<br>Several<br>In the future<br>Recently	It means<br>Were<br>But<br>Have been<br>Over here<br>It is<br>Come on<br>With<br>Things<br>Special<br>Down<br>One more time<br>The fact that<br>To say	This<br>Always<br>Declared<br>As<br>Also<br>All of them<br>Described<br>On<br>More<br>Together<br>Other<br>But<br>Others	Announced<br>Mr<br>In care of<br>Below<br>However<br>BBC<br>The inside<br>Description<br>If<br>etc<br>Quantity	Regarding<br>Come on<br>Reached<br>Internal<br>Situation<br>Similarly<br>Even<br>In common<br>It becomes<br>They become<br>Various<br>Right here	Who<br>Things<br>Behind<br>Saying<br>When<br>Right now<br>On top<br>He spoke<br>Saying<br>He said<br>Among them<br>So	Remembered<br>However<br>It was said<br>And<br>Saying<br>Important<br>Place<br>Explained<br>She was<br>As well<br>Turn it on<br>In<br>Below
