# Supplementary material for: Deep learning-based idiomatic expression recognition for the Amharic language
Source: PLoS One. 2023 Dec 14;18(12):e0295339. doi: 10.1371/journal.pone.0295339 (PMC10720994; doi:10.1371/journal.pone.0295339)
Supplement: Attachment — Submitted filename: Response to Reviewers.docx [file pone.0295339.s002.docx]

**Rebuttal Letter**

Dears,

We were pleased to have an opportunity to revise our manuscript, now entitled “Deep Learning-Based Idiomatic Expression Recognition for the Amharic Language” (PONE-D-23-14519). We have carefully considered the reviewers’ comments and suggestions in the revised manuscript. As instructed, we have attempted to succinctly explain changes made in reaction to all comments. We reply to each comment in a point-by-point fashion. We have track changed the revised manuscript. The responses to the concerns raised by reviewers are below and color-coded as follows: a) Comments from editors or reviewers are shown as *text* in italic font b) Our responses are shown as text.

The reviewers’ comments were very helpful overall, and we appreciate such constructive feedback on our original submission. After addressing the issues raised, we feel the quality of the paper is much improved.

Sincerely

Demeke Endalie

***Editor’s comments***

1. *“There are more than four thousand idioms in Amharic writing system”. May be “in the Amharic language”*

**Answer:** Thank you very much. Corrected as per your valuable suggestion.

2. *Normalization of orthography needs to be better explained. Is substantiality purely phonetic? Is it similar to what we found in Arabic or Hebrew?*

**Answer:** Thank you very much. The substitution of those Amharic characters is based on the their phonetic. it is similar with that of other semitic language families such as Arabic and Hebrew.

3. *“We eradicate stop words” ‘eradicate’ is not a suitable word here, use ‘eliminate’ or ‘remove’. Please explain why do you need to remove stop words for idiom recognition? Why do you need to replace numbers – this should be explained and motivated.*

**Answer:** Thank you very much. We correct as per your insightful suggestion. In this study, stop words are terms that do not contribute to the semantics of the given idiomatic phrase but are used to fill the grammatical structure of the idiomatic statement. Stop words are eliminated to save computational time wasted in processing them. To obtain the feature vectors of the numbers' names, we use the FastText embedding and substitute numbers with their alphabetic characters. In the pre-trained FastText-based word embedding, only Amharic alphabetic characters are employed. As a result, for this research, numbers must be translated to their alphabetic names.

4. *Algorithm 1 is not needed. You can just say you load load vector for each word from the database. But what does it mean “we create a vector of both idiomatic and non-idiomatic(literal) words? It that before the training? Or is that a description of the training? Please elaborate this statement.*

**Answer:** Thank you very much. We removed the algorithm from the manuscript and we replace it with ‘Each idiomatic expression's embedding is constructed using pre-trained FastText embedding. The embedding is then saved as a matrix in which the rows reflect the number of idiomatic expressions in the dataset, and the columns represent the embedding dimension used by the embedding technique. The embedding is built before splitting the dataset into training and testing sets.’

5. *For section 4: who are the idiomatic and non-idiomatic sentences are distributed among training and validate and testing set? Is there any reason why you did not use cross-fold validation.*

**Answer:** Thank you for your very much. We did not employ k-fold cross-validation to assess the performance of the proposed model because the dataset is little and a portion of it is used for model evaluation, reducing the amount of data available for training the model indirectly. We used unique idiomatic and non-idiomatic terms (those not included in the training set) to validate and evaluate the proposed idiomatic recognition model. We tune the hyperparameters using a grid search strategy to train the proposed CNN model. The training, validating, and testing sets are separated after producing the dataset's word2vec of each idiomatic expression. There are 1360, 168, and 172 instances of idiomatic expressions used in training, validating, and testing, respectively. The non-idiomatic expression training, validation, and testing examples are 1280, 160, and 160, respectively.

6. *What features were used for the knn, Randomforest and SVM classifiers? Please explain components of those models for your data? For example, did you use knn over average embeddings?*

**Answer:** Thank you very much. We explain the components of those learning models go with our data in Table 5 of the revised manuscript.

7. *Some suggested references*

**Answer:** Thank you very much. We included those references as per your suggestion as reference [4] and [5].

***Reviewer’s comments***

**Reviewer #1:**

1. *the authors claim in the abstract that idioms are used to conceal information. Reference to support this claim?*

**Answer:** Thank you for your insightful suggestion, and comment. Corrected as per your valuable suggestion. Replace this judgmental sentence to ‘Idiomatic expressions are built into all languages and are common in ordinary conversation. Idioms cannot be deduced immediately from the word from which they are derived. Previous studies reported that idiomatic expression affects many Natural language processing tasks in the Amharic language.’

2. *“With testing and training datasets, the proposed model achieves an accuracy of 80% and 98%, respectively” What does it mean? Do you evaluate the training data?*

**Answer:** Thank you for your insightful suggestion, and comment. We employed an 80 by 10,10 to train, validate, and test-splitting ratio to evaluate the proposed idiomatic recognition model. The proposed model's learning accuracy across the training dataset is 98%, and the model achieves 80% accuracy on the testing dataset.

3. *I’d suggest translating examples*

**Answer:** Thank you very much. We have translated all examples written in Amharic.

4. You probably don’t mean that the total numbers of idioms in all Semitics languages is 4000, but it reads this way in” Amharic is one of the languages grouped under the sematic language families that have more than 4000 idiomatic expressions [3]”.

**Answer:** Thank you very much. We paraphrase the statement as Amharic is one of the languages grouped under the Semitic language families [3]. The Amharic language contains more than 4000 idiomatic clauses (expressions).

5. Many typos/slopy writing, e.g., the authors of [7]—lower case?

**Answer:** Thank you very much. We tried to fix all those errors as much as possible in the revised manuscript.

6. It would be nice to understand what stop words were removed (may be added an appendix). Also, why do you normalize the numerals? Why is it important?

**Answer:** Thank you very much. In this study, stop words are terms that do not contribute to the semantics of the given idiomatic phrase but are used to fill the grammatical structure of the idiomatic statement. Stop words are eliminated to save computational time wasted in processing them. To obtain the feature vectors of the numbers' names, we use the FastText embedding and substitute numbers with their alphabetic characters. In the pre-trained FastText-based word embedding, only Amharic alphabetic characters are employed. As a result, for this research, numbers must be translated to their alphabetic names. The stop words eliminated in this manuscript are shown in the appendix section.

7. Section 3.3 –the evaluation metrics are pretty standard as well. It not a criticism, but I am not sure if the authors need a separate section for it.

**Answer:** Thank you very much. We add section 3.3 as a part of section 4.

8. *main criticism of this paper is that the paper doesn't tell a story. The paper briefly mentioned the dataset (but doesn't really describe it), the representation, experiments, evaluation, and numbers. So, what does it all mean? How hard is the task? What kind of idioms did you deal with? Explain your data collection and annotation. How did you obtain your dataset? Who annotated the data? What's the inter-rater agreement? Are there expressions that can appear idiomatically and literally depending on the context? I.e., do you have the same PIEs (potentially idiomatic expressions) that appear in both the idiomatic and non-idiomatic training/test set?*

**Answer:** Thank you very much. We have rewritten the dataset description as per your valuable comments and suggestions. The book writers already annotate the data as idiomatic or literal. The idiomatic terms used in this study were hand-picked and cross-checked from published books. We added Table 1 to support the description of the dataset in the revised manuscript. The data collection for the study was very difficult since we collected them from printed books. The study deals with an idiom having lengths between 2 and 4. We used unique idiomatic and non-idiomatic terms (those not included in the training set) to validate and evaluate the proposed idiomatic recognition model. Related to experimental results and discussion we add descriptions related the what does mean those numbers. For example, the 80% means that the proposed idiomatic detection approach correctly detected 266 idiomatic and non-idiomatic phrases out of 332 cases in the testing dataset. The proposed idiomatic recognition model fails to recognize the remaining 66 testing instances. The results show that deep learning with FastText embedding can detect Amharic idiomatic expressions in a text with better results across multiple quality criteria. This means that researchers or application developers will utilize deep learning-based idiomatic expression identification models to improve the performance of their models or applications.

9. *The experiments are pretty standard. The most interesting part here is the Amharic language and the potential to explore the specific challenges of Amharic, and why the CNN model with FastText representations outperforms the rest. How do you compare with the other approaches to idiom detection? None of the previous approaches is implemented and applied to the Amharic data for comparison. It would be really interesting to see how well they perform on this language. None of it is explored in the paper.*

**Answer:** Thank you very much. We compare these models’ detection accuracy with the proposed model using our dataset with the hyperparameters adjusted, as shown in Table 5. The results show that the proposed algorithm enhanced detection accuracy by 8%, 12%, and 4%, respectively, compared to Random Forest, KNN, and SVM. This is since (1) employing FastText to generate word vectors can yield important features, and (2) processed text features using CNN can better represent high-level characteristics in the given idiomatic and non-idiomatic phrases.

10. *I would start with showing idiomatic expressions in Amharic in context and explain why they present a challenge for the current NLP models. What contribution does this paper make?*

**Answer:** Thank you very much. The main contribution of this study is to develop deep learning plus context-aware word embedding to detect Amharic idioms. We try to present NLP models for comparison purposes.

11. *A lot of missing references. To mention just a few: Sporleder et al., Flor et al., Liu et al., Salton, etc. etc.*

**Answer:** Thank you very much. We add more references as per the requirement.

Thanks very much to the editor and reviewers for your precious suggestions！
