# Supplementary material for: Deep learning-based idiomatic expression recognition for the Amharic language
Source: PLoS One. 2023 Dec 14;18(12):e0295339. doi: 10.1371/journal.pone.0295339 (PMC10720994; doi:10.1371/journal.pone.0295339)
Supplement: Attachment — Submitted filename: Response to Reviewers.docx [file pone.0295339.s003.docx]

**Rebuttal Letter**

Dears,

We were pleased to have an opportunity to revise our manuscript, now entitled “Deep Learning-Based Idiomatic Expression Recognition for the Amharic Language” (PONE-D-23-14519R1). We have carefully considered the reviewers’ comments and suggestions in the revised manuscript. As instructed, we have attempted to succinctly explain changes made in reaction to all comments. We reply to each comment in a point-by-point fashion. We have track-changed the revised manuscript. The responses to the concerns raised by reviewers are below and color-coded as follows: a) Comments from editors or reviewers are shown as *text* in italic font. b) Our responses are shown as text.

The reviewers’ comments were very helpful overall, and we appreciate such constructive feedback on our original submission. After addressing the issues raised, we feel the quality of the paper is much improved.

Sincerely

Demeke Endalie

Comments and their corresponding answers or reflections

*1.
Page 1: "We proposed a conventional neural network (CNN)" You probably mean* *'convolutional', not 'conventional'.*

**Answer:** Thank you very much for your correction. We corrected the spelling as per your suggestion.

*2.
Page 1: "Idiomatic expression is a collection of words that have a different meaning."
Idiomatic expression is not just a 'collection' of words. Consider maybe this formulation:
"Idiomatic expression is a phrase/expression whose meaning may be different from the combination of literal meanings of its composing words. ".*

**Answer:** Thank you very much. Your formulation is better than the former definition, so we took it directly.

*3.
Paragraph that has the sentence "Idiom token classification involves determining if a phrase is literal or idiomatic [4]." and the paragraph starting with "The paper by [6] presents the use of Skip-Thought Vectors". Those two paragraphs use two different references [4] and [6] but essentially refer to the same paper. In fact, reference [4] is correct, but [6] is wrong. Please rewrite the paragraph and eliminate references [6]. Also, do not presume that readers know what Skip-Thought Vectors are, you need to provide an explanation of that term.*

**Answer:** Thank you very much. We include the explanation of skip-thought vectors: “Skip-thought vectors are generated using an encoder-decoder model. After receiving the training sentence, the encoder creates a vector. Encoders include uni-skip, bi-skip, and combine-skip, with uni-skip reading the text from beginning to end, bi-skip concatenating forward and backward results, and combined-skip concatenating vectors.” we eliminate reference [6].

*4.
Regarding the paragraph about reference [9], including the sentence: "Idiom detection as lexical outliers does not make use of class label information." Your description of that work does not make it clear what class labels might be involved and why they are relevant or not. Please make your description clearer.*

**Answer:** Thank you very much. Corrected as per your valuable suggestion. We paraphrased the paragraph as “In [8], a model for detecting idiomatic phrases in written text was proposed. This paper presents a binary classification approach for identifying idioms at the sentence level, offering insights into contexts and unique properties. The authors aim to improve detection rates using textual cohesion and compositionality measures. Textual cohesion refers to the grammatical and semantic relationships between phrases or pieces of a text that contribute to unity and coherence. Textual compositionality measurements, on the other hand, assess the compositional aspects of a text. They used principal component analysis for idiom detection, linear discriminant analysis for discriminant subspace generation, and three nearest neighbor classifiers to obtain accuracy. They also analyzed the advantages and disadvantages of each technique, which are broader than previous idiom identification algorithms.”

*5.
Paragraph starting with the sentence: "Idiomatic expression in language has a detrimental impact on NLP task performance [10]." The problem is that reference [10] discusses the importance of idioms for English language learners (humans), and not for NLP systems. Please revise your paragraph.*

**Answer:** Thank you very much. We rephrase it as Idiomatic expression in language has a detrimental impact on improving language learning proficiency and NLP task performance [9,10].

*6.
Section 2.1, This section talks about CNN, but has reference [11] (García-Laencina et al.), which is a reference to a paper about KNN. Please adjust your references.*

**Answer:** Thank you very much. Corrected and now it has ref no [12].

*7.
Section 2.4 Random Forest Classifiers. This section includes the sentence "To achieve diversity among basic decision trees, random forest chose the randomization approach, which works well with bagging or random subspace methods [15]". However, reference [15] (Patodkar and Sheikh) is not about random forest classifiers, rather it is about Twitter sentiment classification. Please refine your references.*

**Answer:** Thank you very much. Corrected as per your valuable suggestion ref[16] in the revised manuscript.

*8.
Section 3. Materials and Methods This section begins with the sentence, "This study focuses on developing a deep learning model using FastText..." It would be nice to tell the readers t this point what FastText is. Although that is explained later in the manuscript, it would be good to provide a hint at this point in the narrative. Maybe say "...using FastText embeddings...".*

**Answer:** Thank you very much. “FastText is an open-source text representation and categorization framework developed by Facebook's AI Research (FAIR) team. Using the FastText word vector representation, we generate a vector for each word in the corpus that can be directly fed into any learning algorithm. The goal of this study is to develop a deep learning model that uses FastText to detect the presence of idiomatic words in an Amharic text.”

*9.
Section 3.1. Dataset. The description of the dataset is unclear. The sentence "We received 1700 idiomatic words..." is strange, as you are handling expressions – so maybe you meant "1700 idiomatic expressions". Next, the manuscript says "we also collect 1600 phrases that are not classified as idiomatic expressions". Here is the crucial question: what is the nature of those 1600 phrases? Are those phrases that could be idiomatic (in some other context), do those phrases* *overlap with the set of 1700 idiomatic expressions? (one would expect so!) or are the 1600 expressions just some literal expressions that are unrelated to the idiomatic subset ?*

*It is very important to clarify this point. If the expressions in the set of 1700 and those in the set of 1600 are not the same, then we are not talking about idiom token classification.
See for example Adewumi et al. (2021),* [*https://aclanthology.org/2022.lrec-1.72/*](https://aclanthology.org/2022.lrec-1.72/)

**Answer:** Thank you very much. The study does not focus on idiomatic token classification but rather on whether a particular expression is idiomatic or literal by keeping the semantic relationship. We paraphrase the unclear statement as “In this study, we used 3300 isolated Amharic idiomatic and non-idiomatic expressions to train and evaluate the proposed model. Out of 3300 Amharic phrases, 1700 were idiomatic expressions, and 1600 were phrases containing terms found in idiomatic expressions but not utilized as an idiom. The idiomatic phrases were compiled from the aforementioned publications and are easily readable in the books themselves. The 1600 non-idiomatic phrases were collected from those publications.”

10.
*Another clarification point about the dataset: what about the contexts of those collected expressions? Did those expressions appear in sentences? It is not clear whether you classified just isolated expressions or expressions in context (in sentences). This must be clarified and explained explicitly, in detail. You might want to provide some examples from your sets of idiomatic and non-idiomatic cases (with English translations). If you were classifying expressions in sentences, you need to provide some statistics – e.g. what was the average word-count per sentence in each of the subsets (idiomatic and non-idiomatic). Please also indicate the average word-counts per subset after the cleaning and normalization procedures.*

**Answer:** Thank you very much. We used an isolated expression, and we used the following paragraph to clarify the nature of idiomatic and non-idiomatic phrases in the dataset. “Let us use an example to clarify the distinction between idiomatic and non-idiomatic phrases in the dataset.” እግረ ደረቅ ነው” (igire derek’i newi), is an idiomatic phrase which has the meaning "the feet are dry" when we interpret the meaning of each word, but the actual meaning of the phrase is "Unlucky." ” እግረ አባጣ ነው” (igirē ābat’a newi) is a non-idiomatic phrase which means “he has elephantiasis.” This is the only meaning that can be derived from the literal meanings of each word in it. Despite the fact that both phrases utilize the same word, "እግረ," which means leg, the first phrase does not refer to its real meaning, leg, whereas the second phrase does. We use text processing activities such as stop word removal to remove terms such as "ነው” (newi) that have no bearing on the mood of the statement.”

*11.
Regarding section 3.2. It is not clear how are you using the word embeddings. You get an embedding for each word – and then what? You need to describe how the different algorithms use the embeddings! You cannot just say 'embeddings' and then proceed to results. Describe how are the features and/or embeddings integrated in your CNN, KNN, SVM and RF classifiers. This is is the most substantial comment for this manuscript.*

**Answer:** Thank you very much. Corrected as per your valuable suggestion. For CNN “To feed the training, validating, and testing data to the CNN model, we first create three matrices for the training, validation, and testing sets, each with dimensions of (number of training samples,300), (number of validating samples,300), and (number of testing samples,300). The number 300 is chosen for the matrix because the dimension of the matrices we build must be equal to the embedding matrix value of the CNN, as mentioned in Table 4 above. Next, using looping, fill the above matrix with the vector values of the FastText embedding model's idiomatic and non-idiomatic words. Following that, we train the CNN model by supplying the training data matrix and training data label and validate its performance by passing the validation set's matrices with the hyperparameter values shown in Table 4.”

For Machine learning algorithms: “The vector generated by the pre-trained FastText retained the meanings of each idiomatic and non-idiomatic phrase. This vector for the training and testing set, along with their label, is passed to the machine learning algorithms employed in this study, as explained in section 4.1.”

*12.
Regarding TF-IDF, please describe which corpus was used for obtaining the TF and IDF values.*

**Answer:** Thank you very much. Corrected as per your valuable suggestion. “Document Frequency (TF-IDF) and one-hot encoding vectors. IDF =Log [(# Number of documents) / (Number of documents containing the word)] and TF = (Number of repetitions of a word in a document) / (# of words in a document). The TF-IDF is calculated for each of the 3300 Amharic phrases used in this study. To produce our dataset's TF-IDF matrix, we first create a list of unique word lists and then compute the TF-IDF of each word in each phrase. The generated matrix has a dimension equal to the entire number of phrases in the dataset multiplied by the total number of unique words. We append the label of each phrase in the matrix to the end of each row. Then, we divide this matrix into training and testing and train each of the models used in this study.”


*13.
Just after Table 7, there is a sentence "The suggested model generally detected Amharic idioms with acceptable accuracy..." Whether accuracy of 80% is acceptable or not, is debatable. Please avoid self-congratulatory remarks.*

**Answer:** Thank you very much. We paraphrase the sentence as “By combining the benefits of CNN with the pre-trained FastText embedding model, the proposed approach detected Amharic idioms with higher accuracy than other machine learning models and words' vector representation”.
*14.
Section 5 "Conclusion" includes a sentence "This misleads models since..." That is a very awkward phrasing in English. Models are not 'misled'. Models can be inaccurate or produce inaccurate results. Regarding bibliographic references:*

**Answer:** Thank you very much. Corrected the sentence as “Models that do not take idiomatic recognition into account may produce incorrect results since the actual meaning of the expression differs from the meaning of each word that makes up the expression.”

15.
"[2] Oktay Yağiz, "anguage, Culture, Idioms, and Their Relationship with the Foreign Language,"..."L" is missing before "anguage".

**Answer:** Thank you very much. Corrected by adding the missed letter “L”.

16.
"[23] Ton Van der Valk, Jan H. Van Driel, Wobbe De Vos, "Common Characteristics of Models in Present-day Scientific Practice," Research in Science Education, vol. 37, no. 4, pp. 469-488, 2020."
The year of publication is 2007, not 2020.

**Answer:** Thank you very much. Corrected as per your comment, and it becomes ref[24] in the revised manuscript.
