# Supplementary material for: Deep learning-based idiomatic expression recognition for the Amharic language
Source: PLoS One. 2023 Dec 14;18(12):e0295339. doi: 10.1371/journal.pone.0295339 (PMC10720994; doi:10.1371/journal.pone.0295339)
Supplement: Attachment — Submitted filename: Response to Reviewers.docx [file pone.0295339.s004.docx]

**Rebuttal Letter**

Dears,

We were pleased to have an opportunity to revise our manuscript, now entitled “Deep Learning-Based Idiomatic Expression Recognition for the Amharic Language” (PONE-D-23-14519R2). We have carefully considered the reviewers’ comments and suggestions in the revised manuscript. As instructed, we have attempted to succinctly explain changes made in reaction to all comments. We reply to each comment in a point-by-point fashion. We have track-changed the revised manuscript. The responses to the concerns raised by reviewers are below and color-coded as follows: a) Comments from editors or reviewers are shown as *text* in italic font. b) Our responses are shown as text.

The reviewers’ comments were very helpful overall, and we appreciate such constructive feedback on our original submission. After addressing the issues raised, we feel the quality of the paper is much improved.

Sincerely

Demeke Endalie

1. *In the abstract: “We collected 1700 idiomatic and 1600 non-idiomatic clause datasets from Amharic books.” Please delete the words 'clause datasets' and replace them with* *'expressions'*

**Answer:** Thank you very much. We replace the ' clause datasets' with ‘expressions’.

2. Page 2: “The Amharic language contains more than 4000 idiomatic clauses (expressions).”
   Please rephrase it as “The Amharic language has more than 4000 idiomatic expressions.”

**Answer:** Thank you very much. We rephrase as per what you said.

3. Page 2. Take the sentences “Amharic is one of the languages grouped under the Semitic language families [3]. The Amharic language has more than 4000 idiomatic expressions.” and move them just after “all-natural languages [2].”

**Answer:** Thank you very much. We rearranged it as you stated.

4. Page 2. Before the sentence “Idiomatic expression recognition from a given text plays....”,
   please put a heading “Related work”.

**Answer:** Thank you very much. We added it as per your suggestion.

5. Concerning the notion of 'idiom token' classification (as mentioned in the section about related work). After checking out the train- and test- files that are available on your github site, it seems obvious that your research was focused on classification of phrase types rather than tokens. This means detecting whether a phrase is usually an idiom or usually literal. This is a viable research direction, and is OK, but this needs to be very explicitly stated in the manuscript (in the “Dataset” section). This contrasts with idiom token classification, which tries to decide whether a given phrase in a given context (sentence) is used idiomatically or literally. All the papers cited in your 'related work' section are focused on 'idiom token' detection. You should also cite a work that has focused on 'idiom type' detection; specifically I suggest this one: <https://aclanthology.org/E06-1043/> The distinction between idiom type and idiom token classification must be made very clear in the manuscript.

**Answer:** Thank you very much. We added an introductory sentence to the dataset section(section 4.1 in the revised manuscript):” This study concentrated on detecting Amharic idiom types. This means detecting whether a phrase is usually an idiom or usually literal. The dataset for the study is gathered and processed with this major goal in consideration.”

We included an idiom-type detection paper in the related works.

Afsaneh et al. [6] used the connection between idiomaticity and (in)flexibility to create statistical measures to automatically distinguish idiomatic from literal verb plus noun combinations. VNICs differ in flexibility but contrast with compositional phrases, which are more lexically productive and have a wider range of syntactic forms. Lexical and syntactic flexibility can be used as partial indicators of semantic analyzability and idiomaticity.

An algorithm is proposed for the automatic classification of idiomatic and literal expressions [7]. It hypothesizes that high-ranking words in a text segment are less likely to be part of an idiomatic expression. The algorithm uses Latent Dirichlet Allocation (LDA) to extract topics from paragraphs containing idioms and literals. Idiomatic expressions are treated as semantic outliers, and outlier detection is used to distinguish idioms from literals using local semantic contexts.

6. Page 2. Instead of “This paper uses Skip-Thought Vectors...”, please write “Salton et al. [4] used Skip-Thought Vectors...”

**Answer:** Thank you very much. We updated it as per your insightful suggestions.

7. Page 3. “However, according to the researchers' understanding, no Amharic...” please rephrase as “However, according to our best knowledge, no Amharic...”

**Answer:** Thank you very much for your insightful suggestion. We changed it

8. Section 3.2 “Most idiomatic expressions in books are 2 to 4 in a number of tokens exist,”
   please rephrase it as “Most idiomatic expressions in our source books have 2 to 4 tokens,”

**Answer:** Thank you very much. We changed the former sentence with your formulation.

9. In the sentence “...that have no bearing on the mood of the statement." pleas replace 'mood' with 'meaning'.

**Answer:** Thank you very much. We changed it.

10. Section 3.2 “Encoding is highly required...” please rephrase as “Encoding is extensively required...”

**Answer:** Thank you very much. We rephrase the sentence: “Encoding is extensively required to pass texts as input to different machine learning and deep learning models [24].”

11. Section 5. Conclusion Concerning the sentence “However, due to the magnitude of the data, the model's performance requires improvement.” Please rephrase as “Potentially, the model's performance could be improved by training on more data.”

**Answer:** Thank you very much. We rephrase the suggested sentence as per your formulation.

12. “Additional information from the holy books of Amharic...” This is not quite clear, remember that the readers probably know little about this. Please clarify.

**Answer:** Thank you very much. We rephrase the last two sentences of the conclusion to make them clear: “In the future, other datasets from Amharic holy books, such as the Amharic Bible, will be included in the aforementioned model to improve its performance. Furthermore, we propose to use this model as a component in Amharic machine translation.”

13. Please remove any funding-related text from the manuscript and let us know how you would like to update your Funding Statement.

**Answer:** Thank you very much. We removed the funding statement from the manuscript and we submitted it on the online submission.

14. *New references added as related works in the revised manuscript*

| [6]. | Afsaneh Fazly, Suzanne Stevenson, "Automatically Constructing a Lexicon of Verb Phrase Idiomatic Combinations," in 11th Conference of the European Chapter of the Association for Computational Linguistics, Trento, Italy, 2006. |
| --- | --- |
| [7]. | Jing Peng , Anna Feldman, Ekaterina Vylomova, "Classifying Idiomatic and Literal Expressions Using Topic Models and Intensity of Emotions," in Proceedings of the 2014 Conference on Empirical Methods in Natural Language Processing (EMNLP), Doha, Qatar, 2014. |

Thank you so much for providing suggestions on how to improve the quality of our manuscript!
